# Prediction and Mitigation of H_2_S-Induced High-Temperature Corrosion in a 660 MW Boiler Water-Wall

**DOI:** 10.3390/ma19102074

**Published:** 2026-05-15

**Authors:** Jie Liu, Yifan Gu, Lele Feng, Di Yang

**Affiliations:** 1 Key Laboratory of Low Grade Energy Utilization Technology & System of Ministry of Education, School of Energy and Power Engineering, Chongqing University, Chongqing 400044, China; 2 School of Safety Engineering, China University of Mining and Technology, Xuzhou 221116, China; 3 Material Corrosion and Protection Key Laboratory of Sichuan Province, Zigong 643000, China; 4 Shenyang Rundian Heating Power Co., Ltd., Shenyang 110014, China

**Keywords:** utility boiler, H_2_S corrosion model, computational fluid dynamics, water-wall, near-wall air

## Abstract

**Highlights:**

- An assessment method for HTC is developed by coupling H_2_S concentration with wall metal temperature.
- The effects of 3%, 5%, and 7% RFG-NWA ratio on HTC are investigated.
- It is recommended to take NWA protection measures when the boiler load exceeds 50% THA.

**Abstract:**

The high-temperature corrosion (HTC) caused by H_2_S poses a critical threat to the water-wall of unity boilers. To address this challenge, the present work develops a predictive corrosion depth model that integrates two critical determinants: the local concentration of H_2_S and the temperature of the water-wall metal. The proposed methodology is applied to evaluate HTC risks under three distinct thermal loads: boiler maximum continuous rating (BMCR), 75% turbine heat acceptance (THA) and 50% THA. Furthermore, the protective effect of near-wall air (NWA) ratio injection using recirculated flue gas (RFG) was numerically investigated, to quantify their influence on both HTC mitigation and in-furnace combustion characteristics. Key findings indicate that at BMCR load, elevated sidewall temperatures combined with H_2_S enrichment produce a peak corrosion depth of 33.7 μm. At 50% THA, the peak H_2_S concentration drops sharply to 150 ppm, and the corresponding corrosion depth falls to only 7 μm. Consequently, it is recommended that NWA protection measures be implemented whenever the boiler load exceeds 50% THA. Even at a 7% NWA ratio, the impact on the furnace temperature field remains negligible. Meanwhile, it significantly reduces the corroded area and halves the peak corrosion depth, confirming that RFG-based NWA offers a flexible and effective engineering solution for mitigating HTC in coal-fired utility boilers.

## 1. Introduction

As clean energy adoption and carbon abatement gather pace globally, coal-fired power plants remain essential to secure a steady power supply, damping grid fluctuations driven by the intermittency and fluctuation of wind and solar generation [1,2,3]. Technological innovations integrating new and fossil energy sources, such as solar-aided coal-fired power generation, also show attractive performance in terms of energy efficiency and carbon emission reduction [4]. In particular, China’s resource endowment, characterized by abundant coal reserves but limited oil and natural gas, dictates that coal-fired power will continue to serve as a core safeguard for energy supply in the medium and long term [5,6]. Among the emissions from pulverized coal-fired boilers (PC boilers), CO_2_ and NO_x_ are the primary gases of concern [7,8,9,10,11,12], with NO_x_ emissions posing significant environmental risks necessitating effective control measures. Common strategies to reduce NO_x_ formation during combustion include low-NO_x_ burner retrofitting, air-staged combustion, and fuel-staged combustion [13]. Air-staged combustion, which works by dividing the combustion air into primary and secondary streams to create a fuel-rich zone in the lower furnace, effectively suppresses peak flame temperatures and thus curtails thermal NO_x_ formation [14]. Despite its effectiveness in NO_x_ control and cost advantages, this approach induces a reducing atmosphere in the furnace. Under oxygen-limited reducing conditions, the sulfur in coal is more likely to react with hydrogen to form H_2_S instead of oxidizing to SO_2_ [15]. This conversion to H_2_S is detrimental because it accelerates high-temperature corrosion (HTC) of the boiler’s water-walls: H_2_S reacts with the metal surfaces of the water-walls at elevated temperatures, causing gradual material loss, reduced wall thickness, and diminished structural strength. For large-scale utility boilers, this corrosion significantly increases the probability of tube rupture, ultimately restricting the boiler’s ability to operate safely and efficiently.

ISO 15156-1, ISO 15156-2 and ISO 15156-3 offer guidelines for material selection in H_2_S-containing service environments [16,17,18]. Several researchers have investigated and discussed the mechanisms and control measures of water-wall HTC. According to Xiong et al. [19], corrosion products of water-walls exhibit a three-layered structure. The inner layer is derived from the reaction of iron oxide with H_2_S, the intermediate layer from fly ash accumulation, and the outer layer from FeS gas condensation and deposition onto the water-wall. Furthermore, the corrosion rate is also associated with structural factors. Research by Xu et al. [20] has demonstrated that steel materials with a higher surface roughness corrode more rapidly in environments containing SO_2_ and H_2_S. Via simulation methods, Wang et al. [21] examined the water-wall temperature features of a 660 MW opposed-firing PC boiler during continuous load increase and decrease over 30–100% load. Ma et al. [22] studied the combined HTC of NH_3_-H_2_S on water-wall materials under a strong reducing atmosphere in ammonia-coal co-firing. It is observed that NH_3_ reduces Fe_2_O_3_ to Fe_3_O_4_/Fe, which easily reacts with H_2_S to produce iron sulfides. NH_3_ also degrades such sulfides, increasing the H_2_S concentration pressure in the corrosion layer. Yuan et al. [23] proposed an H_2_S corrosion model to predict material loss on water-walls in the lower furnace. The model accounts for three critical independent parameters: metal temperature, H_2_S concentration and exposure duration. Using experimental corrosion data for pure Fe and SA213-T2 boiler steel, the predictive model is formulated as a mathematical equation through regression analysis. Measures to mitigate HTC include using alloy tubing resistant to high-temperature sulfide corrosion, controlling sulfur and alkali metal contents in feed coal, and optimizing air distribution to increase local oxygen concentration [24,25,26,27]. Near-wall air (NWA) serves as an efficient approach to optimize air distribution. It generates an oxygen-rich air layer that insulates against hot gases while oxidizing CO and H_2_S. Numerical simulations by Zheng et al. [28] indicated that NWA significantly reduced ash deposition intensity in the primary combustion zone, which in turn weakened HTC hazards on water-walls. In addition, the Analytic Network Process was utilized to optimize the location of NWA nozzles. Huang et al. [29] conducted CFD simulations to explore combustion and corrosion properties under the synergistic influence of NWA arrangement and combustion optimization. It was revealed that increasing the NWA ratio from 2.5% to 4.5% markedly raises the O_2_ volume fraction in high-corrosion-risk areas.

Flue gas recirculation represents an advanced combustion technology widely utilized in power plant and industrial boiler applications. Recirculation fans are utilized to reintroduce high-temperature flue gas from the tail flue into the furnace, which brings about prominent advantages such as better homogeneity of furnace heat load, lower NO_x_ emissions, and higher main steam temperature during low-load operation. Hu et al. [30] noted that the reduction efficiency rose with both the fuel equivalence ratio and recirculation ratio. Through experiments and simulations, Zhang et al. [31] demonstrated the NO_x_ emission reduction performance of pulverized coal reburning when synergistically modified with syngas and recycled flue gas. According to Ling et al. [32], wet recycled flue gas strengthened combustion characteristics in biomass oxy-fuel conditions, yet marginally reduced convective heat transfer in the heat exchanger. Our previous investigations effectively integrated flue gas recirculation with NWA technology, demonstrating the merits of employing recycled flue gas as the source for NWA [33]. In practical engineering applications, the volume of recycled flue gas typically accounts for 10–20% of the total flue gas flow. Unlike NWA supplied from conventional secondary air, any proportion above 5% tends to disrupt the organization of air-staged combustion. Utilizing recycled flue gas as NWA can further enhance the NWA effect by increasing its flow ratio while avoiding the adverse impact on low-load combustion stability caused by injecting recycled flue gas in a single region.

The key aspects of this study are summarized as follows: (1) Previous work typically gauged corrosion severity using only CO or H_2_S concentrations, neglecting the metal wall’s significant impact on corrosion behavior. To address this, a comprehensive numerical model for HTC prediction is proposed, integrating H_2_S concentration and wall metal temperature. (2) Because coal-fired utility boilers are now crucial for deep peak regulation in power grids, load changes simultaneously impact the in-furnace atmosphere, working fluid flow, and wall heat flux distribution. Corrosion depths were therefore predicted under BMCR, 75% THA, and 50% THA loads. (3) Conventional NWA is derived from secondary air and limited to a 5% ratio, as the nozzle placement between the burner and over-fire air (OFA) means higher ratios would impair air-staging effectiveness. A novel NWA technology employing RFG has been shown to prevent HTC as effectively as conventional NWA, with an RFG recirculation ratio of 10–15% of the total flue gas flow. Consequently, the influence of the RFG-derived NWA ratio on HTC and boiler combustion characteristics was numerically investigated.

## 2. Establishment of HTC Prediction Model of Water-Walls

### 2.1. Overview of HTC Prediction Model

The HTC of boiler water-walls is dominated by sulfide chemical reactions, whose reaction rate follows the Arrhenius equation and is mainly dependent on the reaction temperature and main reactant (e.g., H_2_S) concentration. In investigations into water-wall corrosion, CO, O_2_, and H_2_S concentrations adjacent to the water-wall surface are widely adopted as corrosion indices. HTC risk is regarded as low under weakly reducing conditions with CO < 3% and O_2_ > 2%, or when the H_2_S concentration is less than 200 ppm [33,34,35]. Under combustion tuning or constant-load operation, modifications mainly influence the near-wall gas atmosphere, whereas the water-wall surface temperature changes marginally. Hence, variations in gas composition can sufficiently quantify the corrosion degree. Nevertheless, utility boilers currently play a key role in flexible peak regulation for power systems. Fluctuations in load modify both the furnace atmosphere and the temperatures of the flue gas and working fluid inside the water-wall tubes, which in turn influence the corrosion reaction temperature. Additionally, NWA technology not only mitigates corrosive gas concentrations near the wall but also effectively reduces the wall temperature. Consequently, relying solely on atmospheric conditions is insufficient to comprehensively assess the impacts of load variation and NWA injection on HTC. In practical operation, both corrosion control performance and operational cost must be balanced to reduce the water-wall corrosion rate to a critical threshold. Currently, there is a lack of quantitative corrosion risk criteria to guide the prevention and control of HTC. Hence, this work combines a CFD model, hydrodynamics calculations approach, and a zero-dimensional heat transfer model of the water-wall surface.

**Figure 1:**
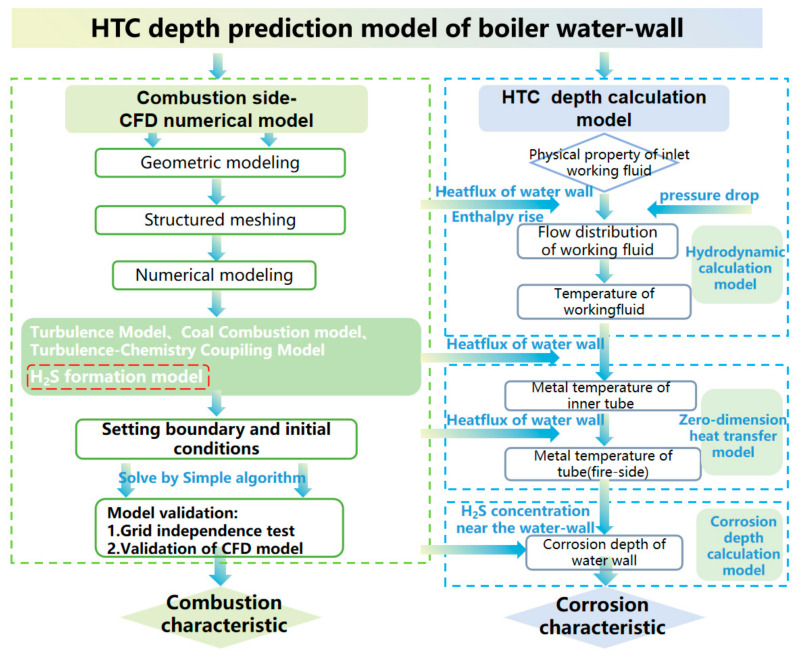
Flowchart of the HTC depth prediction model for boiler water-walls.

Based on the H_2_S concentration and metal temperature on the fire side of the water-wall, the corrosion depth of the water-wall surface was calculated, enabling accurate prediction of HTC damage under various load conditions. This criterion was also adopted to optimize the NWA technique. The establishment process of the water-wall HTC prediction model is shown in Figure 1.

### 2.2. CFD Numerical Model of Boiler

**Figure 2:**
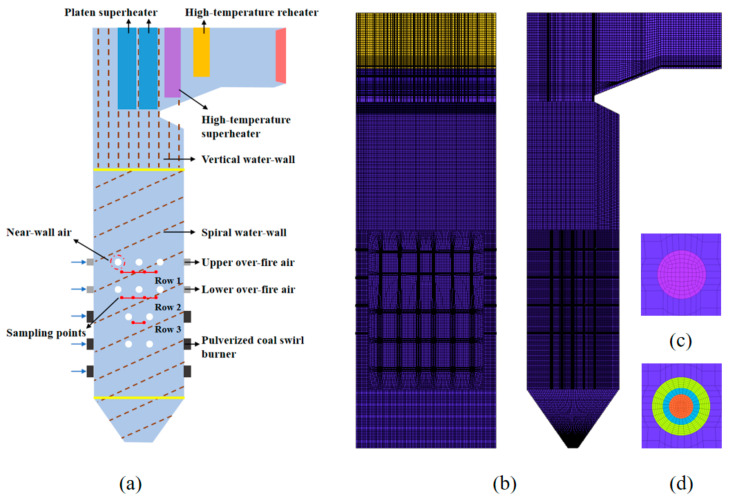
Schematic diagrams of (**a**) boiler computational domain, (**b**) grid system, (**c**) surface grid of OFA, (**d**) surface grid of swirl burner.

**Figure 3:**
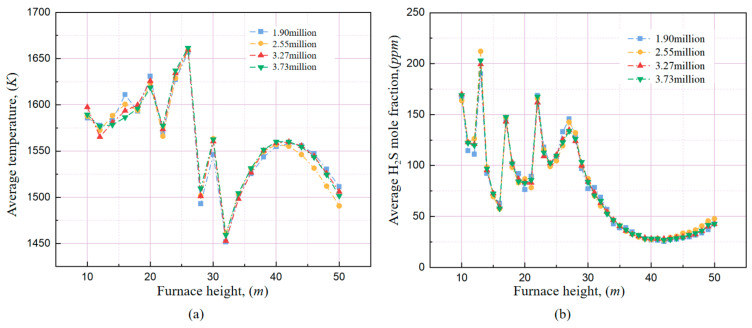
Grid independence verification: (**a**) temperature; (**b**) H_2_S mole fraction.

A 660 MW ultra-supercritical opposed-firing PC boiler is selected as the modelling object. The computational domain of this boiler is displayed in Figure 2a. This boiler was symmetrically equipped with 20 side-wall NWA nozzles, 36 swirl coal combustors and 24 over-fire air nozzles. The numerical mesh of the boiler furnace is shown in Figure 2b–d, the different colours represent different boundary part. Based on the results of the grid independence test, as shown in Figure 3, the grid system with 3.27 million grid cells was selected for the numerical calculations in this study. The default post-calculated sulfur revolution model in the commercial CFD software Fluent is based on a simplified eight-step species reaction mechanism which ignore the effect of some main combustion species on the formation of H_2_S, such as O_2_, CO, H_2_O, resulting the poor prediction accuracy. In our previous work, a global sulfur species gas-phase reaction mechanism proposed by Zhang et al. [36] was implemented to replace the default mechanism, by using the user-defined function (UDF).

A brief description of the numerical setups is given in Table 1. In this work, CFD calculations are done by commercial CFD software ANSYS-Fluent 2020R2.

Table 2 provides the boundary conditions for different boiler loads in the simulation. Table 3 lists the proximate and ultimate analysis of coal samples.

### 2.3. Validation of CFD Model

**Figure 4:**
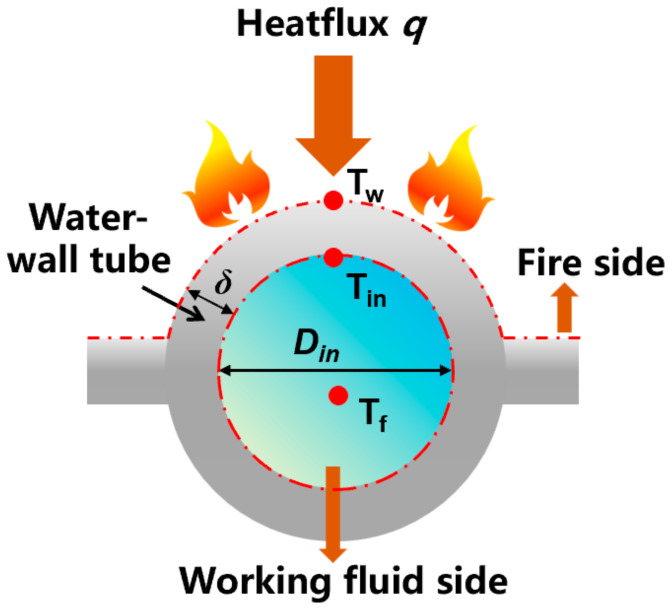
Schematic of heat transfer for water-wall tubes.

Following the unit retrofit, wall flue gas composition measurements were performed at 650 MW high load. Relevant parameters such as heating surface temperature, boiler outlet NO_x_ emission and fly ash combustible content were statistically analyzed. Flue gas at the wall surface on both sides of the water-wall was sampled via on-line flue gas measuring tubes, and the flue gas components were determined by a portable flue gas analyzer. The layout of sampling points is shown in Figure 4. Three layers of flue gas sampling holes were arranged below the NWA nozzles at elevations of 30.3 m, 36.3 m and 39.8 m. Among them, four sampling holes were set on each of the upper two layers, while two sampling points were arranged on the bottom layer; the arrangement of sampling points is shown in Figure 2.

In these field tests, the Testo-Pro 350XL portable flue gas (Testo SE & Co. KGaA, Lenzkirch, Germany) analyzer was used to measure the concentrations of CO, O_2_ and NO_x_. Its measuring range was 10,000 ppm (0–1%) for CO, 200,000 ppm (0–20%) for O_2_, and 1000 ppm for NO_x_. The Testo 350 portable flue gas (Testo SE & Co. KGaA, Lenzkirch, Germany) analyzer was adopted for H_2_S concentration measurement, with a measuring range of 1000 ppm. During the test period, no soot blowing or slag removal operations were performed to avoid fluctuations in key operating parameters. Numerical simulation was conducted under the BMCR condition close to the actual on-site operating state, and the simulation results were compared with measured data, as presented in Table 4. It can be seen that the simulation results are in good agreement with the measured data, which verifies the reliability of the established numerical model.

### 2.4. Calculation Model of Water-Wall Metal Temperature on the Fire Side

#### 2.4.1. Zero-Dimensional Heat Transfer Model for Water-Wall Surfaces

As shown in Figure 4, the metal temperature on the fire side of the water-wall is mainly determined by the heat flux density on the fire side wall surface, q, and the working fluid temperature inside the tube, Tf. The required wall heat flux and working fluid temperature are derived from the CFD numerical model and the hydrodynamic calculation model presented in the following text. According to the different working fluid states in each section, the corresponding convective heat transfer coefficient was used for heat transfer calculation.

Under subcritical single-phase flow, the convective heat transfer coefficient is defined by the Dittus–Boelter formula, which is expressed as Equation (1). The saturated boiling heat transfer coefficient inside inclined tubes is obtained via Equation (2), as referenced in [37]. With regard to smooth surfaces under supercritical conditions, the corresponding heat transfer coefficient is determined by employing Equation (3), as cited in [38].

(1) $$\alpha =0.023\frac{\lambda }{D_{in}}Re^{0.8}Pr^{0.4}$$

(2) $$\frac{\alpha }{\alpha_{l}}=0.0885{\left[{\left(\frac{x}{1-x}\right)}^{0.9}{\left(\frac{\rho_{1}}{\rho_{s}}\right)}^{0.5}{\left(\frac{\mu_{s}}{\mu_{l}}\right)}^{0.1}\right]}^{0.1516}{\left(\frac{p}{p_{cr}}\right)}^{-5.2231}{\left(\frac{G}{2000}\right)}^{-0.1664}$$

(3) $$\alpha =0.0068\frac{\lambda_{b}}{D_{in}}Re_{b}^{0.9}Pr_{b}^{0.63}{\left(\frac{\rho_{w}}{\rho_{b}}\right)}^{0.17}{\left(\frac{\lambda_{w}}{\lambda_{b}}\right)}^{0.29}$$

where λ denotes the thermal conductivity of the working fluid, MW/m-k; $D_{in}$ denotes the inner diameter of a single tube, m; μ denotes the dynamic viscosity, pa/s; *Re* denotes Reynolds number; *Pr* denotes Prandtl number; ρ denotes the density of the working fluid in the saturated state, kg/m^3^. Subscripts *w* and *b* represent the thermophysical properties corresponding to the working fluid temperature and the tube wall temperature, respectively.

Equations (4) and (5) are adopted to compute the inner wall temperature on the fire side of the water-wall tube. Using this temperature as an input, the surface temperature on the fire side (i.e., the metal wall temperature) is then evaluated via Equations (6) and (7) [39].

(4) $$T_{in}=T_{f}+q\cdot \beta /\alpha$$

(5) $$\beta =D_{in}/(D_{in}+2\delta )$$

(6) $$T_{w}=T_{in}+q(\delta /\lambda_{tube})[2\beta /(\beta +1)]$$

(7) $$\lambda_{tube}=-0.023{\bar{T}}_{tube}+52.712,\;673\;K\leq {\bar{T}}_{tube}\leq 873\;K$$

where $T_{f}$ denotes the temperature of the working fluid inside the tube; q denotes the heat flux density of the water-wall towards the fire side; *β* denotes the ratio of the inner and outer diameters of the water-wall tube; δ denotes the thickness of the tube wall, taken as 0.075 m based on the thickness of common water-wall tubes; $\lambda_{tube}$ denotes the thermal conductivity of the tube wall material, which varies linearly with the average tube wall temperature ${\bar{T}}_{tube}$ [38]. An assumption is adopted in the calculations whereby the average tube wall temperature exceeds the temperature of the working fluid by 50 K [40].

#### 2.4.2. Simplified Hydrodynamic Calculation Method

The boiler water-wall investigated in this paper comprises two distinct structural configurations: a spirally ascending tube section in the lower region and a vertically ascending tube section in the upper region. This arrangement is commonly adopted in high-parameter PC boilers. Compared with vertical water-walls, spiral water-walls facilitate uniform heat flux distribution and enhance heat transfer efficiency, thereby effectively preventing local overheating and exhibiting superior adaptability to the high-temperature and high-pressure conditions of supercritical and ultra-supercritical PC boilers. Consequently, spiral water-wall tubes are usually arranged in the main combustion zone of the boiler.

**Figure 5:**
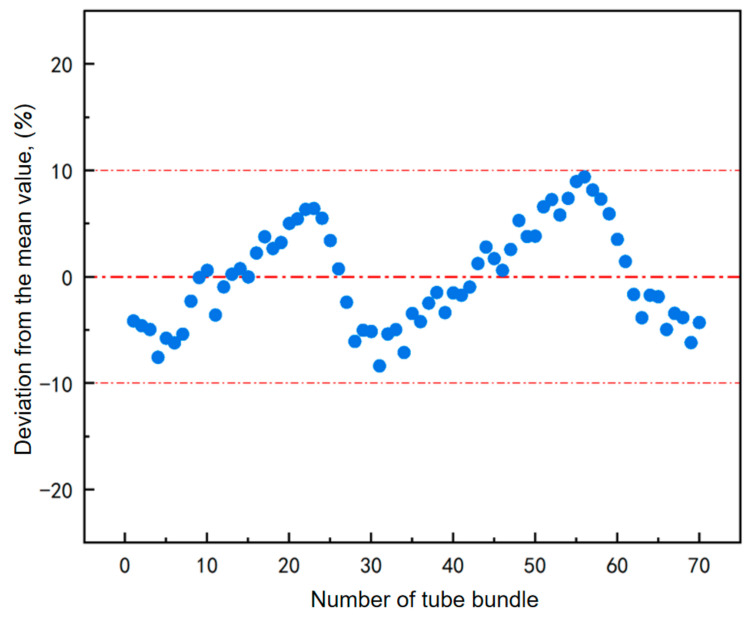
Error analysis of heat flux input using average heat flux from all tubes.

Owing to the excellent and efficient heat transfer performance of the spiral water-wall tubes, the axial heat flux distribution trends along the seventy tubes demonstrate similar patterns [41]. A comparative error analysis between the heat flux of an individual tube and the mean heat flux of the seventy tubes is presented in Figure 5. The maximum error remains within 10%, indicating that the total heat fluxes across the seventy tubes may be regarded as approximately uniform. Consequently, to achieve a reasonable simplification in the calculation of working fluid temperature, it is assumed that the temperature distribution of the working fluid within all tubes is identical, and the mean heat flux of the entire tube bundle is employed for calculating the working fluid temperature in a single representative tube [42].

**Figure 6:**
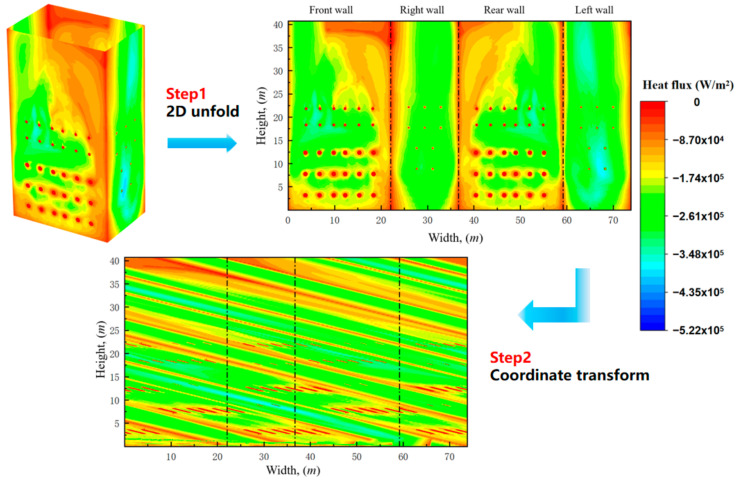
Heat flux reallocation method of CFD output heat flux data.

**Figure 7:**
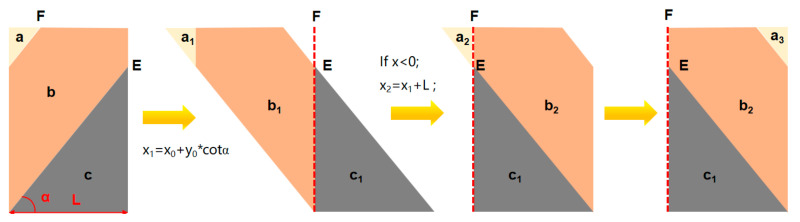
Schematic diagram of coordinate transform method.

For spiral water-wall tubes, the wall heat flux is not allocated along the working fluid flow direction within the tube bundle when the fluid temperature and tube wall temperature are computed via conventional approaches. Yuan et al. [43] pointed out that calculating the temperature using a heat flux distribution along the wall height would underestimate the actual metal temperature of the water-wall, which would affect the risk prediction of the water-wall. In contrast, the metal temperature of water-walls was obtained by adopting a heat flux reallocation model proposed by Yuan et al. [23], which along the flow direction of the tube showed good agreement with field-measured data. Therefore, the heat flux reallocation model was adopted to accurately calculate the metal temperature in this work; the process diagram of the heat flux reallocation model d is shown in Figure 6. And coordinate transform method is shown in Figure 7, the dashed line “EF” represents the flow direction of the working fluid inside the spiral water wall tube, and the regions in different colors represent the processing batches during the coordinate transformation process.

Accordingly, the following approach is adopted for heat flux redistribution, whereby the heat flux density is imposed on the tube wall along the flow direction of the tube bundle, as shown in Figure 4. First, the heat flux distribution at the spiral water-wall of the four walls (front, rear, and two side walls) is obtained via CFD numerical simulation. Given that the heat absorption in the hopper section is limited and exerts a minor effect on the temperature rise of the working fluid in the water-wall, the hopper region is neglected in the calculation, and the inlet of the water-wall tubes is considered to be located below the four walls. Subsequently, the heat flux density data of the four walls are subjected to two-dimensional unfolding, followed by coordinate transformation of the unfolded heat flux distribution along the inclined direction of the water-wall, so that the heat flux is distributed along the flow direction of the working fluid inside the tubes.

Based on the wall heat flux distribution obtained from CFD calculations, the along-path heat flux distribution of the water-wall tubes is further derived using the aforementioned heat flux redistribution model. A single water-wall tube is divided into 40 small segments along its flow path. With the known inlet pressure and thermophysical properties of the working fluid, the enthalpy rise due to heat absorption and the pressure drop caused by flow in each segment along the flow direction are calculated step by step, yielding the thermophysical parameters, thermodynamic state and steam pressure of the working fluid inside each tube segment. Subsequently, the corresponding convective heat transfer coefficient is selected according to the varying fluid state in each segment, and the fireside metal temperature of the tube wall is finally determined via a zero-dimensional wall heat transfer model.

The pressure drop within water-wall tubes is determined using the approach described below. The total pressure drop of the working fluid flowing through these tubes comprises two components, namely the frictional pressure drop *p_f_* and the gravitational pressure drop *h_f_*, which are given by Equation (8).

(8) $$\Delta p(i,M_{i})=\sum_{j}\Delta p_{f}(i,j,M_{i})+\sum_{j}\Delta p_{h}(i,j,M_{i})$$

With regard to smooth tube walls, the corresponding single-phase pressure drop is determined according to Equation (9).

(9) $$\Delta p_{f}=f(L/D_{in})(G^{2}/2)\nu$$

where *f* denotes the single-phase pressure drop under turbulent flow conditions in smooth-walled tubes. *G* denotes the mass flow rate of the working fluid in kg/s, which can be calculated via Equation (10). With reference to the commonly used water-wall tube material SA213-T12, the surface roughness parameter *k* in the equation is specified as 0.008.

(10) $$f=\frac{1}{4{\left[\mathrm{lg}(3700D_{in}/k)\right]}^{2}}$$

For the steam-water two-phase mixture, the corresponding pressure drop is computed by means of Equation (11) [44].

(11) $$\Delta p_{f}=\psi f(L/D_{in})(G^{2}/2)\nu_{1}[1+x(\nu_{s}/\nu_{1}-1)]$$

where ψ denotes the friction correction coefficient, which is determined by the value of *G* and calculated via Equation (12).

(12) $$\begin{array}{l} \psi =1,\;\mathrm{if}\;G=1000\;\mathrm{kg}/(m^{2}\cdot s)\\ \psi =1+\frac{x(1-x)\frac{\rho_{1}}{\rho_{s}}(\frac{1000}{G}-1)}{1+x(\frac{\rho_{1}}{\rho_{s}}-1)},\;\mathrm{if}\;G\;<\;1000\mathrm{kg}/(m^{2}\cdot s)\\ \psi =1+\frac{x(1-x)\frac{\rho_{1}}{\rho_{s}}(\frac{1000}{G}-1)}{1+(1-x)(\frac{\rho_{1}}{\rho_{s}}-1)},\;\mathrm{if}\;G>\;1000\;\mathrm{kg}/(m^{2}\cdot s) \end{array}$$

For single-phase flow conditions, the gravitational pressure drop is computed by means of Equation (13).

(13) $$\Delta p_{h}=\rho gh$$

In the scenario of a water-steam two-phase flow mixture, the gravitational pressure drop is computed by adopting Equation (14).

(14) $$\Delta p_{h}=[xp_{s}+(1-x)\rho_{1}]gh$$

where *h* refers to the height of the working fluid inside the inclined tube, with its precise calculation predicated on the mathematical formulation presented in Equation (15).

(15) h=L×sinα

where *L* denotes the length of the tube wall, and α denotes the inclination angle of the spiral water-wall tube.

The aforementioned calculations of water-wall hydrodynamics and fireside temperature were performed using MATLAB R2023b.

### 2.5. Prediction Model for Metal Corrosion Depth of Water-Walls

As stated in the introduction, HTC is induced and dominated by chemical corrosion. Numerous experimental investigations have validated that the HTC rate of water-walls follows the Arrhenius law. To achieve an effective quantitative assessment of HTC risks, researchers have reproduced various realistic corrosion environments in experiments, calculated the corrosion depth on steel surfaces, and determined the influences of metal temperature, reactant concentration and tube materials on the corrosion rate. Kung [45] conducted HTC experiments on Fe-based materials with various Cr contents under different H_2_S concentrations and wall temperatures. An empirical correlation for calculating the HTC rate, as presented in Equation (16), was proposed based on three key parameters: the H_2_S concentration in the wall flue gas, the wall temperature, and the Cr content in the wall steel.

(16) $$R_{cor}^{y}=8128\times \exp(-\frac{15818}{1.987T_{w}})\times {\left[C_{H_{2}S}\right]}^{0.574}\times \frac{1}{(10.5+\omega_{Cr}{)}^{1.234}}$$

where $R_{cor}^{y}$ represents the annual corrosion rate, measured in mm/year. The units for H_2_S concentration, wall temperature and Cr content are ppm, K and %, respectively.

Pronobis and Litka [46] performed a comparative analysis of the HTC kinetic experimental results reported by Davis et al. [47] and Nakagawa et al. [48]. For specific steel materials, they concluded that the HTC rate is primarily correlated with wall temperature $T_{w}$, CO concentration in the flue gas $C_{CO}$, and the inherent chlorine content in coal $C_{Cl}$. They then proceeded to quantify the relationship between these parameters and the HTC rate in a pilot plant, developing the empirical correlation for HTC rate given by Equation (17).

(17) $$R_{cor}^{h}=C_{\mathrm{CO}}\times (17.91C_{CO_{\max}+7.63})\times (1.82\times 10^{-6})Tw^{2.224}\times (14.64C_{\mathrm{Cl}}^{2}-0.67Cl+1.01)$$

where $R_{cor}^{h}$ represents the corrosion rate per hour, with a unit of nm/h. The units for CO concentration, wall temperature and chlorine content in coal are %, K and %, respectively.

Similarly, Xu et al. [49] conducted an experimental investigation into the corrosion behavior of 12Cr1MoV, a commonly used material for water-walls, under field-measured wall atmosphere and tube temperature conditions in boilers. It was found that under fixed corrosive conditions, the corrosion rate follows a parabolic correlation with corrosion time, indicating that the growth rate of corrosion diminishes as time increases. Structural analysis of the corrosion layer suggests that the corrosion layer thickness continuously increases under laboratory conditions, thereby reducing the corrosion rate. Even under a constant corrosive environment, the corrosion rate is not a constant value. Consequently, the effect of corrosion time on the corrosion process should be taken into account in HTC prediction. Meanwhile, in actual boilers, the corrosion layer can spall due to external mechanical forces, returning the corrosion process to the initial rapid corrosion stage, which results in lower corrosion rates derived from laboratory tests compared with those in practical boiler operation.

Yuan et al. [23] experimentally simulated the corrosion effects of various water-wall atmospheres on T22 steel, a commonly used water-wall material. Corrosion kinetic data were obtained by varying the H_2_S concentration and steel surface temperature, and a prediction model for the corrosion depth of water-wall tubes was proposed by incorporating corrosion time, as shown in Equation (18). Field validation demonstrated that the model exhibits high accuracy. The maximum deviation of corrosion depth predicted by this empirical formula from laboratory data is 4.3%, with its applicable H_2_S concentration limit below 800 ppm. The H_2_S concentration range adopted in this paper is less than 620 ppm, which satisfies the applicable scope of the empirical formula. Nimmervoll et al. [50] utilized a tube furnace to reproduce the actual corrosive atmosphere of water-wall tubes and compared the corrosion resistance of austenitic steels with different elemental compositions under the coexisting corrosion environment of HCl and H_2_S. The results show that chromium in steel plays a vital role in resisting sulfide corrosion, whilst nickel can effectively withstand chloride corrosion. The water-wall material of the object boiler is 12CrMoVG. The corrosion model proposed by Yuan et al. [23] was originally established for T22 steel, which corresponds to the Chinese national standard grade 12CrMoG. The most significant difference between the two steels lies in a 1% vanadium alloying element content, while their remaining chemical compositions are almost identical. Vanadium primarily improves the toughness and strength of steel, as well as its resistance to hydrogen corrosion. Accordingly, the calculation error arising from applying this model to 12CrMoVG is considered to be within an acceptable range.

(18) $$R_{cor}^{D}={\left[22.023\times C_{H_{2}S}\times \exp(-\frac{9886}{T_{w}})\times t\right]}^{0.693}$$

where $R_{cor}^{D}$ is the corrosion depth, measured in μm; $C_{H_{2}S}$ is the concentration of H_2_S in the flue gas, measured in ppm; *T_w_* is the metal temperature on the fire side of the water-wall, measured in K; *t* is the corrosion time, measured in h.

In this work, the corrosion depth calculation model proposed by Yuan et al. [23] is adopted. The H_2_S concentration near the wall and wall heat flux are obtained via CFD numerical simulations, and the metal temperature on the fire side of the wall is derived from hydrodynamic calculations combined with a zero-dimensional wall heat transfer model. The wall corrosion depth under various operating conditions is finally determined, allowing quantitative prediction and assessment of HTC risks.

## 3. Results and Discussion

### 3.1. HTC Under Different Loads

**Figure 8:**
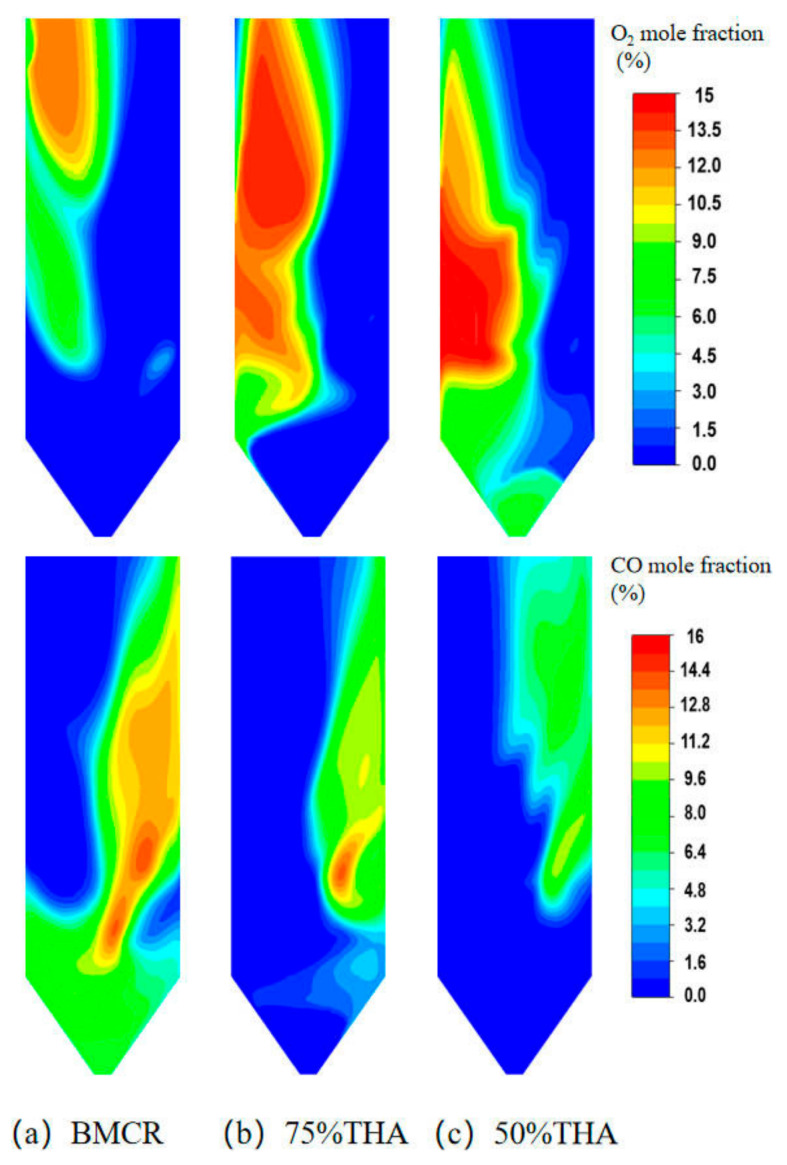
Distribution of O_2_ and CO concentration near the sidewall under different boiler loads: (**a**) BMCR load, (**b**) 75% THA load, (**c**) 50% THA load.

Figure 8 shows the distributions of CO and O_2_ near the sidewall under different loads. It can be observed that CO and O_2_ are distributed in opposite zones, displaying a competitive pattern. Furthermore, the concentrations of CO and O_2_ on one sidewall are asymmetrically distributed under different load conditions. With decreasing load, the excess air coefficient of the furnace increases, leading to a gradual rise in wall O_2_ concentration, accompanied by a decrease in CO concentration and a continuous reduction in the area of the CO distribution region. When the boiler load is reduced from the boiler maximum continuous rate (BMCR) to 75% turbine heat acceptance (THA), the O_2_ concentration on the left sidewall increases significantly, the region with CO concentration above 10% shrinks obviously, and the CO concentration at the ash hopper drops below 4%. Nevertheless, a small area still exhibits CO concentration exceeding 12%. With a further load reduction to 50% THA, the excess air coefficient increases further, and the coal mill operation mode of the boiler is altered, with the lower-layer burners shut down. The O_2_ concentration in the lower furnace rises distinctly, the reducing atmosphere adjacent to the wall is greatly improved, and the CO concentration is uniformly reduced to below 8%.

**Figure 9:**
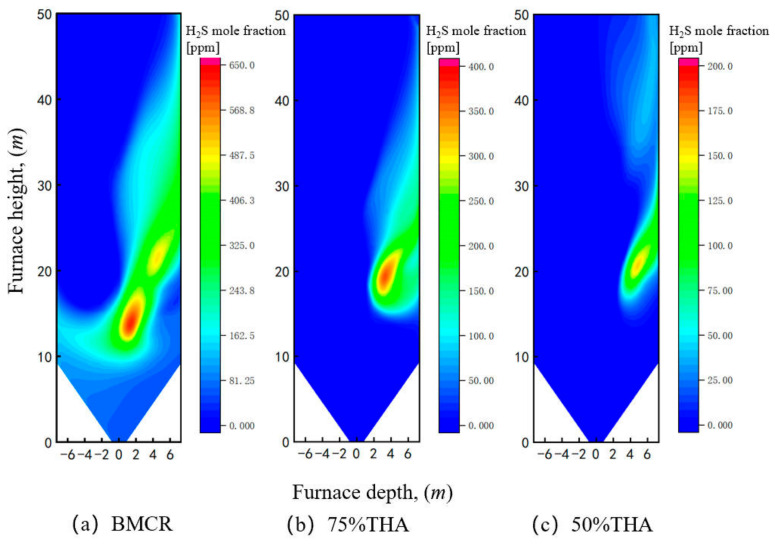
Distribution of H_2_S near the sidewall under different boiler loads: (**a**) BMCR load, (**b**) 75% THA load, (**c**) 50% THA load.

Figure 9 shows the distribution of H_2_S concentration near the sidewall under different loads. As can be seen, the H_2_S concentration distribution exhibits a strong correlation with that of CO. The reason is that under a reducing atmosphere, CO reduces FeS_2_ to FeS with simultaneous generation of H_2_S, while CO also forms COS, which is a precursor of H_2_S [33,51]. Consequently, the sidewall H_2_S concentration decreases with decreasing boiler load, and the distribution region gradually shifts towards the burner side. This phenomenon may be attributed to the reduction in inlet air velocity at lower loads, which weakens the scouring effect of unburned carbon on the central region of the sidewalls. At BMCR load, the peak H_2_S concentration on the wall is approximately 620 ppm, with concentrations in most H_2_S distribution regions ranging from 200 to 400 ppm. At 75% THA load, the H_2_S distribution range is significantly reduced, and the peak concentration drops to about 360 ppm. At 50% THA load, the peak H_2_S concentration is further markedly reduced to approximately 150 ppm, accompanied by a substantial decrease in distribution area.

**Figure 10:**
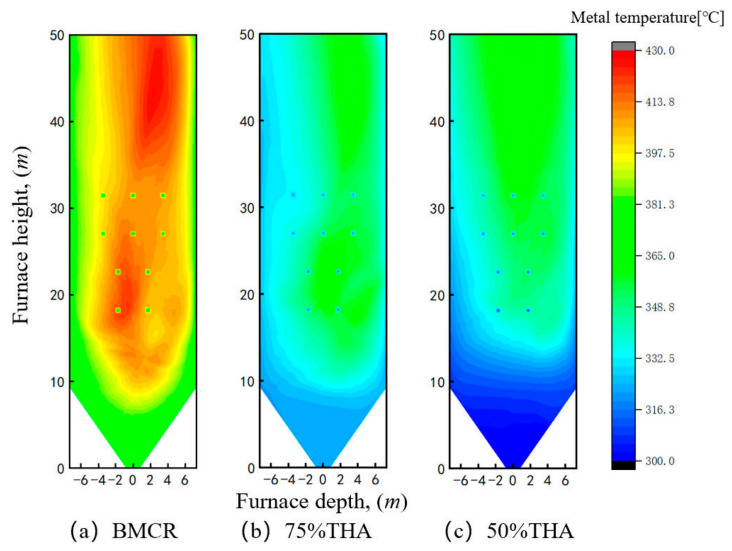
Metal temperature distribution of left sidewall under different boiler loads.

Using the wall temperature calculation method described in Section 2.3, the wall temperature distributions of the left sidewall under various loads were obtained, as presented in Figure 10. In general, the high-temperature zones on the sidewall are concentrated in the central region, and the wall temperature gradually increases with height. This is because the working fluid temperature rises continuously along the flow direction, and the combustion organization of opposed flames leads to the strongest flue gas radiative heat transfer in the central area. Under BMCR load, the wall temperature is approximately 360 °C in the hopper and part of the main combustion zone below 15 m; in the height range of 15–34 m, local high-temperature zones reach about 400 °C; above 34 m, most of the wall temperature exceeds 400 °C, with the central region at around 420 °C. As the load decreases to 75% THA, the overall wall temperature drops significantly, with the average temperature falling from 387 °C to 341 °C. The temperature reduction in the upper high-temperature zone reaches 60 °C. When the load is further reduced to 50% THA, shutting down the lower burners results in minor changes in the heat flux of the middle and upper sections but obvious variations in the lower section. The temperature in the hopper and part of the main combustion zone below 15 m decreases further from 330 °C at 75% THA to about 305 °C, leading to an average wall temperature of 337 °C. Overall, the wall temperature decreases with decreasing load. On the one hand, a lower load reduces the coal feed rate into the furnace, thus decreasing the total heat absorption of the wall. On the other hand, as the load decreases, the inlet temperature of the working fluid inside the water-wall drops from 339 °C at BMCR to 316 °C and 298 °C at 75% THA and 50% THA, respectively. Combined with the reduced heat absorption of the wall, the temperature rise of the working fluid along the water-wall also decreases. It is also noteworthy that different mill operation modes in actual operation exert a significant influence on the wall metal temperature distribution.

**Figure 11:**
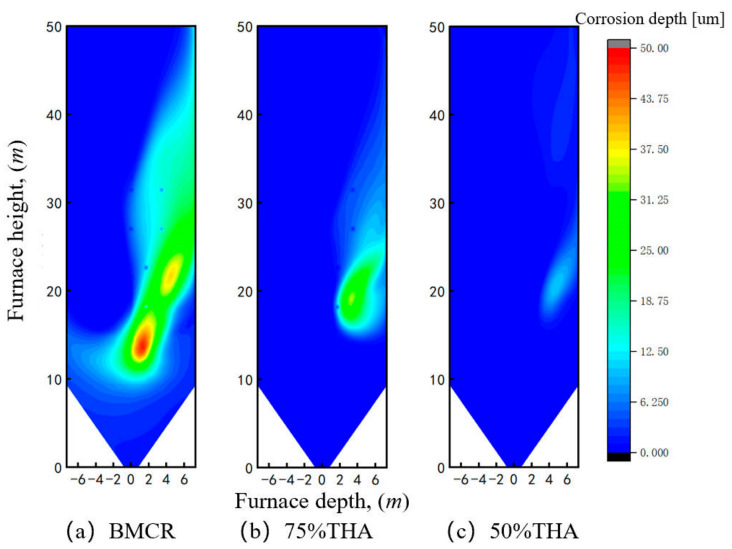
HTC depth distribution of sidewall under different boiler loads.

Based on the corrosion depth prediction model proposed in Section 2.4 above, the wall corrosion depth of the target boiler under continuous operation for 720 h was calculated. The wall corrosion depth under different loads is shown in Figure 11. Under BMCR load, the overall temperature difference in the H_2_S concentration distribution zone is relatively small, so the corrosion depth distribution is close to that of H_2_S concentration. The wall temperature is generally lower in the lower part and higher in the upper part. Affected by the wall temperature, the corrosion depth in the lower wall region with similar H_2_S concentration is shallower than that in the upper wall region. When the load is reduced to 75% THA, both the wall temperature and the peak H_2_S concentration decrease, and the area of the high H_2_S concentration zone shrinks, resulting in a reduction in the peak corrosion depth and the corresponding region. When the load is reduced to 50% THA, both the wall temperature and H_2_S concentration are at low levels. Overall, the wall HTC risk is alleviated with decreasing load. The peak wall corrosion depth and the area of the corrosion region exceeding 5 μm under different loads were statistically analyzed for quantitative evaluation of wall corrosion, as listed in Table 5. Under BMCR load, the most severe corrosion depth reaches 33.7 μm, which decreases to 23.8 μm at 75% THA and further to 7.3 μm at 50% THA. For the corrosion area, regions with a corrosion depth higher than 5 μm are defined as HTC risk zones for statistics. 

The water-wall has a nominal thickness of 7.5 mm. In accordance with power station operating regulations, replacement is required when wall thickness loss exceeds 20% (i.e., 1.5 mm). Considering HTC in isolation, the corrosion depth over a 720 h period is 5 μm, corresponding to an approximate annual corrosion rate of 0.06 mm. The contribution of high-temperature corrosion to overall thickness loss is therefore negligible, and under this criterion, the operating condition is deemed to fall within the HTC safe zone. For the corrosion area, regions with a corrosion depth higher than 5 μm are defined as HTC risk zones for statistics. The total area of the left sidewall surface is 690.54 m^2^. Under BMCR, 29.05% of the wall area is at high-temperature corrosion risk, while the percentages decrease to 8.59% and 1.29% at 75% THA and 50% THA, respectively.

### 3.2. Influence of Recycled Flue Gas NWA Ratios

**Figure 12:**
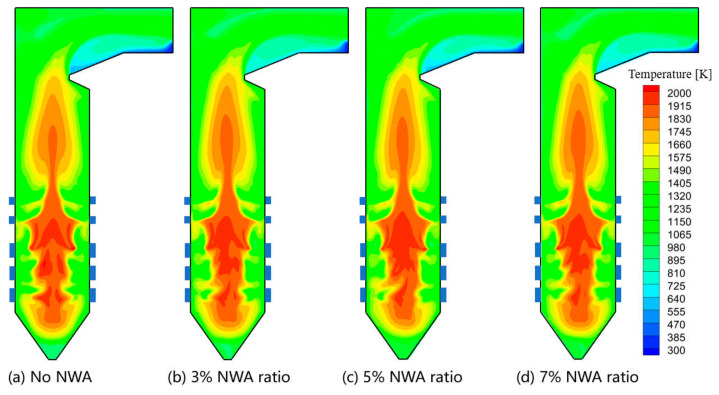
Temperature distribution at center cross-section under different NWA ratios.

The effect of the NWA ratio as sourced from RFG on the boiler combustion characteristics was studied. Figure 12 shows the temperature fields on the central cross-section under various recycled flue gas NWA ratios. It can be observed that increasing the recycled flue gas NWA ratio only slightly affects the overall distribution of the temperature field. Even the injection of NWA at a 7% ratio neither disrupts the air-staged combustion organization in the furnace nor influences the coal combustion process. The effect of recycled flue gas NWA on the peak temperature and the overall temperature field is almost negligible.

**Figure 13:**
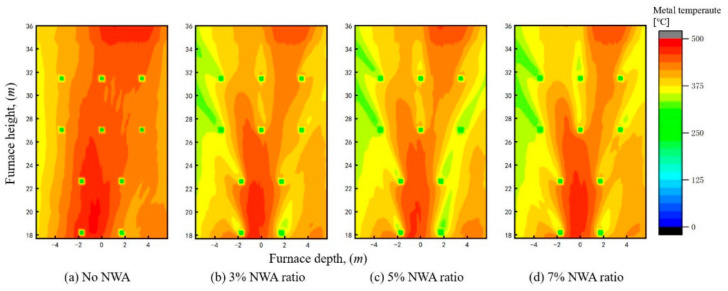
Metal temperature distribution at the sidewall under different NWA ratios.

Figure 13 shows the effect of different recycled flue gas NWA ratios on the metal temperature of the furnace sidewalls. The results indicate that a recycled flue gas NWA ratio of 3% can reduce the local wall metal temperature from 409 °C to 385 °C. As the recycled flue gas NWA ratio is increased to 5%, the magnitude of the wall metal temperature reduction is similar, but the region affected by the cooling effect gradually expands. When the recycled flue gas NWA ratio is further raised to 7%, the average wall temperature in the affected zone remains consistent with that under the 5% wall-air condition, with no further improvement in cooling performance. It is speculated that the increased air volume raises the actual inlet velocity above the design velocity of the NWA nozzles, leading to excessive rigidity of the NWA jet and a higher contribution of velocity vectors towards the furnace center, thereby weakening the dispersion of wall-air along the side walls.

**Figure 14:**
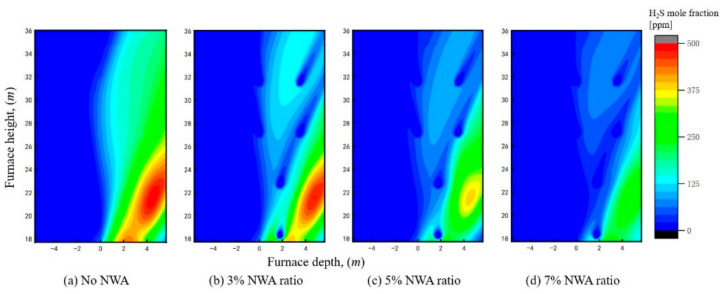
H_2_S concentration distribution at the sidewall under different NWA ratios.

**Figure 15:**
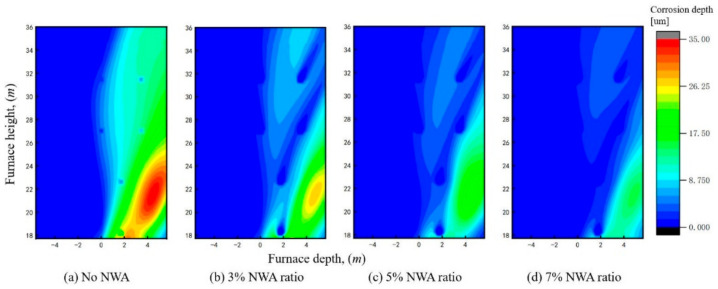
HTC depth distribution at the sidewall under different NWA ratios.

Similarly, statistical analyses were conducted on the H_2_S concentration and corrosion depth on the sidewalls under different NWA ratios, as presented in Figure 14 and Figure 15. It can be observed that the H_2_S concentration exhibits a continuous decreasing trend with an increase in the recycled flue gas NWA ratio. Under the 3% NWA ratio condition, although the H_2_S concentration in most regions has been reduced to below 150 ppm, it remains above 400 ppm in some local areas. However, in terms of corrosion depth, the corrosion depth in these high H_2_S concentration regions is reduced by approximately 26% compared with the case without NWA injection. This indicates that even in regions with high H_2_S concentration, the decrease in metal wall temperature leads to a significant reduction in the corrosion rate. Under the 7% NWA ratio condition, despite no further improvement in the cooling effect on the metal wall temperature, the H_2_S concentration is further reduced, the area of the corroded region is further diminished, and the peak corrosion depth decreases to 15.25 μm, which is about half of the value under the condition without near-wall air.

## 4. Conclusions

A numerical model for predicting the HTC of boiler water-walls is proposed in this work. The model is applied to assess the HTC risk at different operating loads, and the influence of recirculated flue gas NWA ratio on HTC and in-furnace combustion is analyzed. The main conclusions can be summarized as follows:

(1) At BMCR load, a relatively significant HTC risk arises due to the high wall temperature of the sidewalls and the enrichment of high-concentration H_2_S. At 75% THA load, the H_2_S concentration decreases considerably, yet remains at approximately 300 ppm in local regions. Meanwhile, the average wall metal temperature drops by about 40 °C owing to the reduction in input heat load, thereby alleviating HTC to a certain extent. At 50% THA load, the peak H_2_S concentration falls sharply to 150 ppm, accompanied by a substantial decrease in wall temperature in the burner-out layers. As a result, the peak corrosion depth is reduced from 33.7 μm at BMCR to 7.3 μm, leading to a markedly lower high-temperature corrosion risk. In summary, it is recommended to take proactive NWA protection measures when the load exceeds 50% THA.
(2) Numerical investigations on the NWA ratio demonstrate that the NWA ratio using recycled flue gas can exceed the 5% ratio limit associated with conventional secondary air NWA systems. Even at a NWA ratio of 7%, its impact on the in-furnace temperature field distribution remains negligible. Moreover, increasing the NWA ratio progressively enhances the effectiveness of HTC prevention. A higher NWA ratio also allows for the installation of more NWA nozzles, thereby offering a novel engineering solution for the staged introduction of high-proportion recycled flue gas.

## Figures and Tables

**Table 1:** Description of CFD numerical model setups.

Models	Details
Turbulence	Realizable k-ε model
Radiation heat transfer	Discrete ordinate (DO) model
Multiphase flow	Discrete phase model (DPM)
Coal devolatilization	Chemical percolation devolatilization (CPD) model
Char combustion	Diffusion/kinetics model
Interaction of turbulence on chemistry	Presumed probability density function (PDF) model
Solver	Steady double precision pressure-based solver
Pressure-velocity coupling scheme	SIMPLE

**Table 2:** Boundary conditions of different boiler loads.

	Parameters	BMCR	75% THA	50% THA
Mass flow rate (kg/h)	Primary air	4.32 × 10^5^	3.27 × 10^5^	2.38 × 10^5^
	Secondary air	1.11 × 10^6^	8.32 × 10^5^	6.63 × 10^5^
	OFA	7.18 × 10^5^	5.39 × 10^5^	4.29 × 10^5^
Temperature (K)	Primary air	399.79	390.71	375.38
	Secondary air	599.65	579.15	566.15
	OFA	599.65	579.15	566.15
	NWA	618	\	\
Coal mass flow (kg/s)		2.08	1.55	1.00
Excess air ratio		1.224	1.330	1.513

**Table 3:** Proximate and ultimate analysis of coal.

Ultimate analysis/%	C_ar_	H_ar_	O_ar_	N_ar_	S_ar_
	54.76	3.55	4.69	0.98	1.08
Proximate Analysis/%	A_ar_	M_ar_	V_ar_	FC_ar_	
	27.44	7.50	21.31	43.75	

**Table 4:** Comparison between simulated and measured results.

Measurement Metrics	Numerical Simulation Results	Measured Results
Temperature at the lower section of the superheater (K)	1437	1450
Mass flow rate at flue outlet (t/h)	2455.3	2481.7
Average O_2_ concentration at upper measuring points (%)	12.85	11.97
Average O_2_ concentration at mid-level measuring points (%)	8.64	7.79
Average O_2_ concentration at lower measuring points (%)	15.03	14.60
Average CO concentration at mid-level measuring points (%)	0.84	>0.55 *
Average CO concentration at lower measuring points (%)	0.26	0.25

“*” for the average values of measuring points in the table indicates that the CO concentration at some measuring points exceeded the upper range limit of 1%, and such measuring points were calculated at a fixed value of 1% for average evaluation.

**Table 5:** Quantitative analysis results of HTC situation at sidewall.

Load	BMCR	75% THA	50% THA
Peak corrosion depth (μm)	33.7	23.8	7.3
Area of corrosion depth > 5 μm (m^2^)	212.0	62.7	9.4
Proportion of area with corrosion depth > 5 μm (%)	29.05	8.59	1.29

## Data Availability

The original contributions presented in this study are included in the article. Further inquiries can be directed to the corresponding authors.

## Abbreviations

The following abbreviations are used in this manuscript:

BMCR	Boiler maximum continuous rating
HTC	High-temperature corrosion
NWA	Near-wall air
OFA	Over-fire air
RFG	Recirculated flue gas
THA	Turbine heat acceptance
